# Metabolomic Analysis of Phytochemical Compounds from Ethanolic Extract of Lime (*Citrus aurantifolia*) Peel and Its Anti-Cancer Effects against Human Hepatocellular Carcinoma Cells

**DOI:** 10.3390/molecules28072965

**Published:** 2023-03-26

**Authors:** Pakkapong Phucharoenrak, Chawanphat Muangnoi, Dunyaporn Trachootham

**Affiliations:** Institute of Nutrition, Mahidol University, Nakhon Pathom 73170, Thailand; pakkapong.phu@mahidol.ac.th (P.P.); chawanphat.mua@mahidol.ac.th (C.M.)

**Keywords:** food waste, citrus, anti-cancer, PLC/PRF/5 cells, LC-qTOF/MS, GC-MS

## Abstract

Lime peels are food waste from lime product manufacturing. We previously developed and optimized a green extraction method for hesperidin-limonin-rich lime peel extract. This study aimed to identify the metabolomics profile of phytochemicals and the anti-cancer effects of ethanolic extract of lime (*Citrus aurantifolia*) peel against liver cancer cells PLC/PRF/5. The extract’s metabolomics profile was analyzed by using LC-qTOF/MS and GC-HRMS. The anti-cancer effects were studied by using MTT assay, Annexin-PI assay, and Transwell-invasion assay. Results show that the average IC_50_(s) of hesperidin, limonin, and the extract on cancer cells’ viability were 165.615, 188.073, and 503.004 µg/mL, respectively. At the IC_50_ levels, the extract induced more apoptosis than those of pure compounds when incubating for 24 and 48 h (*p* < 0.0001). A combination of limonin and hesperidin showed a synergistic effect on apoptosis induction (*p* < 0.001), but the effect of the combination was still less than that of the extract at 48 h. Furthermore, the extract significantly inhibited cancer cell invasion better than limonin but equal to hesperidin. At the IC_50_ level, the extract contains many folds lower amounts of hesperidin and limonin than the IC_50_ doses of the pure compounds. Besides limonin and hesperidin, there were another 60 and 22 compounds detected from the LCMS and GCMS analyses, respectively. Taken altogether, the superior effect of the ethanolic extract against liver cancer cells compared to pure compound likely results from the combinatorial effects of limonin, hesperidin, and other phytochemical components in the extract.

## 1. Introduction

Liver cancer is the sixth most common cancer in both sexes worldwide. It is the fifth most common cancer in males and the ninth most common cancer in females [1]. The global incidence rate of liver cancer is estimated at 9.5 in 100,000 persons, and the mortality rate is estimated at 8.4 in 100,000 persons in 2020 [2]. It has a poor prognosis and the highest death rate among all types of cancer. The 5-year relative survival of liver and intrahepatic bile duct cancer patients is 20.8% during the years 2012–2018, with an increasing death rate over 30 years [3]. Liver cancer is caused by multiple factors, including hepatitis infection (Hepatitis B and Hepatitis C), non-alcoholic fatty liver disease, or exposure to carcinogens such as aflatoxin, alcohol, tobacco, contraceptives, nitrosamine, etc. The risk factors vary by geographic area. The most common risks of liver cancer worldwide are hepatitis viruses and alcohol consumption [4]. Hepatitis B infection is a major global health problem found in 3% of the population worldwide [5]. One of the most unique characteristics of liver cancer is the hotspot mutation of the p53 gene at arginine in the codon 249th. The most common mutation is the *TP53 R249S* mutant, in which G > T transversion at the third base pair of codon 249 leads to the substitution of arginine by serine [6]. This is associated with aflatoxin B1 exposure and hepatitis B viral infection [6]. Therefore, it is important to identify new anti-cancer agents against liver cancer with the hotspot p53 mutation. Some liver cancer cell line models, such as PLC/PRF/5, are suitable for the study because they secrete hepatitis virus B surface antigen and contain such hotspot mutation [6]. Previous studies have shown that plant extract contains a variety of phytochemicals that may provide anti-cancer effects, i.e., inducing cell cycle arrest, inhibiting cell proliferation, increasing apoptosis, and prohibiting migration, invasion, and metastasis. It is a promising option with low adverse effects [7].

Lime (*Citrus aurantifolia*) is one of the citrus fruits that has been cultivated around the world. In 2019, the global production of lime was about 20,050,000 metric tons [8]. The commonly consumed portion of lime is juice, which accounts for about 48% of its whole weight. The rest (52%) includes the peel, seed, and dehydrated segment, which becomes food waste from lime consumption or the lime juice industry. The waste from lime is over 10,426,000 metric tons per year [8]. Utilizing the by-product for health benefit is an important area of research that not only reduce environmental problems from waste but also supports food security [9]. Interestingly, in many plants, bioactive compounds are more abundant in the unconsumed parts than that in the regularly consumed part [9]. Lime is a source of phytochemicals, including compounds in phenolic acids, flavonoids, and limonoid groups. These compounds can be found in every part of lime fruits, with most rich in peel (flavedo and albedo tissue) [10,11]. Among phytochemicals found in lime peel, hesperidin and limonin are the ones well-documented for their anti-cancer effects. Previous studies found that hesperidin could inhibit cancer cell proliferation and induce apoptosis through reactive oxygen species (ROS)-mediated mechanism. In addition, it was shown to suppress metastasis and angiogenesis of cancer cells via AKT and NF-κB mediated mechanisms [12,13]. Limonin could induce cell apoptosis and suppress the invasion via the Wnt/β-catenin signaling pathway. In addition, limonin could suppress the glycolysis pathway via the promotion of HK-2 activity, resulting in reduced energy generation and cancer cell death [14,15]. Previous studies showed that hesperidin or limonin could inhibit cell proliferation and invasion of liver cancer cells with wild-type p53 [16,17]. Recently, we developed a green extraction method to achieve hesperidin-limonin-rich lime peel ethanolic extract [18]. However, the metabolomics profile of phytochemicals of the lime peel ethanolic extract and its anti-cancer effect on p53-mutated liver cancer cells (PLC/PRF/5) were unknown. 

## 2. Results

### 2.1. LC-qTOF/MS Screening and Identification

**Figure 1:**
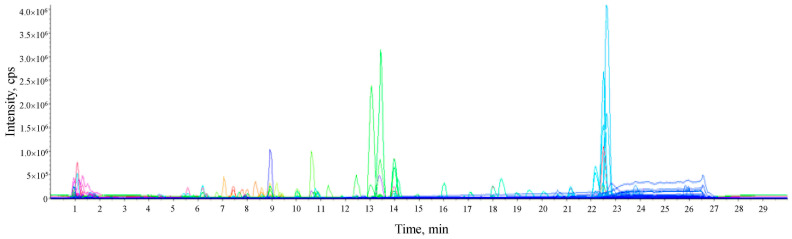
Representative LC-HRMS total ion chromatogram of untargeted metabolome from the ethanolic extract of Lime (*Citrus aurantifolia*) peel. The peaks with high intensity included the purple peak at 8.92 min (hesperidin), the green peak at 13.06 and 13.98 min (5,7-Dimethoxycoumarin and limonin, respectively), and the light blue peaks at 22.51 and 22.6 min (bergaptol and 5-Geranoxy-7-methoxy-coumarin, respectively).

The lime peel ethanolic extract was analyzed by HPLC-qTOF-MS. The untargeted screening and identification of phytochemical components were acquired in positive ion full scan mode and comparisons with mass fragment ion information in the database (library matching score for fit was >70). It resulted in the identification of 62 components (shown in Figure 1 and Table 1). They belong to different chemical groups, which include glycosides, saccharides, amino acids, organic acids, alkaloids, flavonoids, flavonoid glycosides, furanocoumarins, and limonoids. The major components were 7-methoxy-coumarin, 5,7-dimethoxycoumarin, bergaptol, bergamotin, limonin, hesperidin, and neohesperidin. 

### 2.2. GC-HRMS Screening and Identification

**Figure 2:**
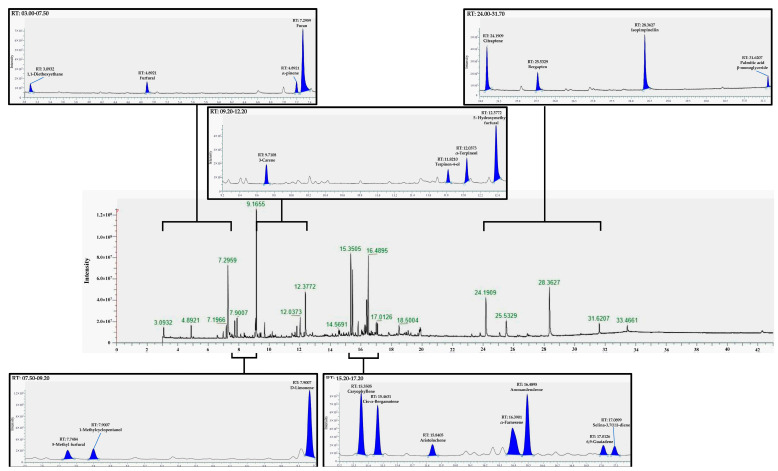
Representative GC-HRMS total ion chromatogram of untargeted metabolome from the ethanolic extract of Lime (*Citrus aurantifolia*) peel.

Chemical constituents of lime peel ethanolic extract were screened and identified using the GC-HRMS method. Figure 2 illustrates the chromatogram of 22 components, the intensity of each compound, the elution properties, and their retention time. Their mass spectra from GC-HRMS analysis were compared with the NIST mass spectra database. The components that have a similarity index greater than 600 are reported in this study. The information on 22 components, including compound name, retention time, molecular mass, molecular formula, similarity index (match factor), and reverse similarity index (Reverse match factor), is shown in Table 2. The major components were D-limonene, furan, aromandendrene, isopimpinellin, caryophyllene, cis-α-bergamotene, and α-farnesene. 

The peaks with high intensity included the peaks at 7.2959, 9.1655, 12.3772, 15.3505, 16.4895, 24.1909, and 28.3627 min, which are furan, D-Limonene, 5-Hydroxymethy furfural, Caryophyllene, Aromandendrene, Citraptene, and Isopimpinellin, respectively.

### 2.3. The Concentration of Hesperidin and Limonin in the Extract

Measured by using LC-MS/MS, the concentrations of limonin and hesperidin in the ethanolic extract are 2.072 and 3.353 mg/g, respectively. The concentration of limonin and hesperidin in the extract that had been dissolved in 0.5% DMSO and treated to the cells at IC_50_ level (503.004 μg/mL) are 0.2868 μg/mL and 0.0139 μg/mL. 

### 2.4. Cytotoxicity on PLC/PRF/5 Hepatoma Cells

**Figure 3:**
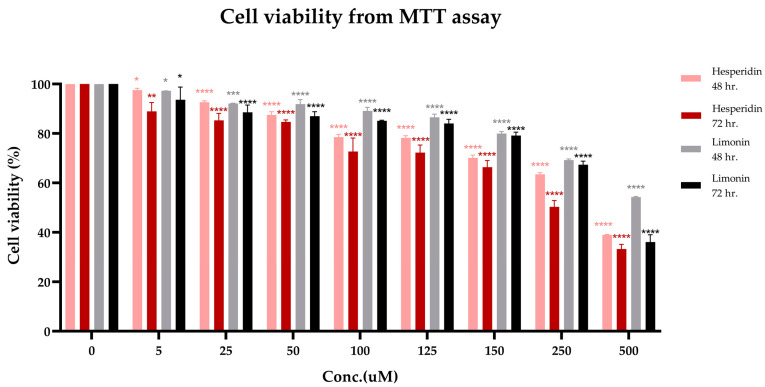
Cell viability (from MTT assay) of PLC/PRF/5 human hepatocellular carcinoma cells treated with hesperidin for 48 h. (pink bars), 72 h. (red bars) and limonin for 48 h. (grey bars), 72 h. (black bars). The statistical differences were separately analyzed for limonin and hesperidin by using one-way ANOVA followed by the Bonferroni comparisons test. The symbol *, **, *** and **** mean *p* < 0.05, <0.01, <0.001 and <0.0001, respectively, compared with the negative control (0.5% DMSO).

The cytotoxicity of hesperidin and limonin on PLC/PRF/5 human hepatocellular carcinoma cells is shown in Figure 3. The cytotoxicity of both compounds showed a significant effect on cell viability starting from 5 μM. This effect was increased with the dose- and time-response relationship. The IC_50_ levels at 72 h of hesperidin and limonin were 271.416 μM (165.615 μg/mL) and 399.713 μM (188.073 μg/mL), respectively. 

**Figure 4:**
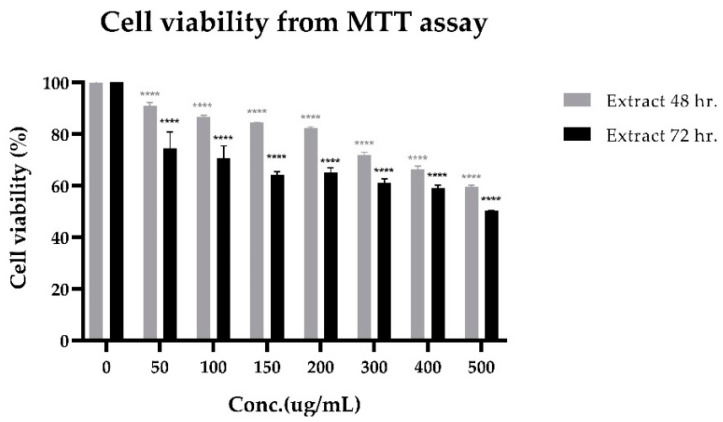
Cell viability (from MTT assay) of PLC/PRF/5 human hepatocellular carcinoma cells treated with lime peel ethanolic extract for 48 h. (grey bars) and 72 h. (black bars). The statistical differences were separately analyzed for limonin and hesperidin by using one-way ANOVA followed by the Bonferroni comparisons test. The symbol **** mean *p* < 0.0001, compared with the negative control (0.5% DMSO).

The cytotoxicity of lime peel ethanolic extract on PLC/PRF/5 human hepatocellular carcinoma cells is shown in Figure 4. A significant decrease in cell viability was observed starting from 50 μg/mL. This effect was increased with the dose- and time-response relationship. It was toxic to PLC/PRF/5 cells, with IC_50_ levels of more than 500 μg/mL. The IC_50_ level estimated from the statistic is 503.004 μg/mL (at 72 h, 95% CI). Upon considering concentrations of compounds only, the concentrations of hesperidin and limonin in lime peel extract at IC_50_ levels are 0.2868 μg/mL and 0.0139 μg/mL, respectively (measured by the method following our previous study [18]). It is 580 and 13,628 times less than the IC_50_ of pure compounds of hesperidin and limonin, respectively. This result suggests that there may be an interaction between bioactive compounds in the extract.

### 2.5. Anti-Invasion Effect on PLC/PRF/5 Hepatoma Cells

**Figure 5:**
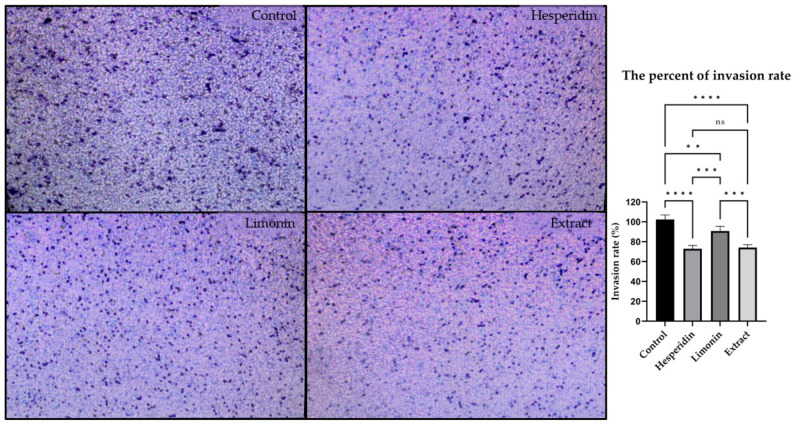
Representative images (Left) and percent invasion rate (Right) of PLC/PRF/5 human hepatocellular carcinoma cells following 0.5% DMSO (negative control), hesperidin, limonin, and lime peel ethanolic extract treatment. The statistical differences were analyzed by using one-way ANOVA followed by the Bonferroni comparisons test. The symbol ns, **, *** and **** mean *p* > 0.05, <0.01, <0.001 and <0.0001, respectively.

The invasion ability of PLC/PRF/5 human hepatocellular carcinoma cells was assessed using Transwell assay. PLC/PRF/5 cells were treated with 10 μg/mL of lime peel ethanolic extract, 0.610 μg/mL (1 μM) hesperidin or 0.470 μg/mL (1 μM) of limonin, (0.5% DMSO was used to negative control). Figure 5 shows the representative images and percent invasion rate of cells after treatment for 24 h. The results showed that PLC/PRF/5 cells could invade through the membrane matrix from the upper well to the lower chamber. It indicated that after treatments, the invasion abilities of PLC/PRF/5 cells were significantly decreased after treatment with hesperidin, limonin or extract when compared with controls. The invasion rates following limonin, hesperidin, and extract treatment were decreased by 9.26 ± 4.73%, 27.14 ± 3.25%, and 25.95 ± 1.51%, respectively. The extract significantly inhibited cancer cell invasion better than limonin but equal to hesperidin.

### 2.6. Induction of Apoptosis in PLC/PRF/5 Hepatoma Cells

**Figure 6:**
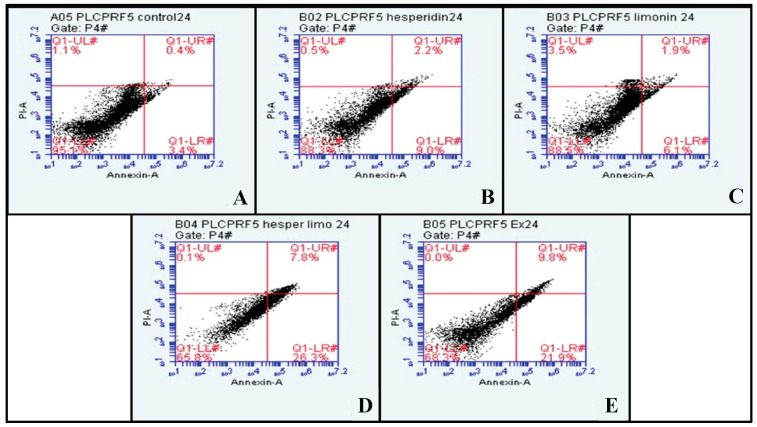
The dot plots of PLC/PRF/5 human hepatocellular carcinoma cells from flow cytometry analysis of Annexin V FITC/PI at 24 h in each treatment are (**A**) negative control (DMSO), (**B**) Hesperidin at IC_50_, (**C**) Limonin at IC_50_, (**D**) Combination of Hesperidin and Limonin at IC_50_, and (**E**) lime peel ethanolic extract peel at IC_50_.

**Figure 7:**
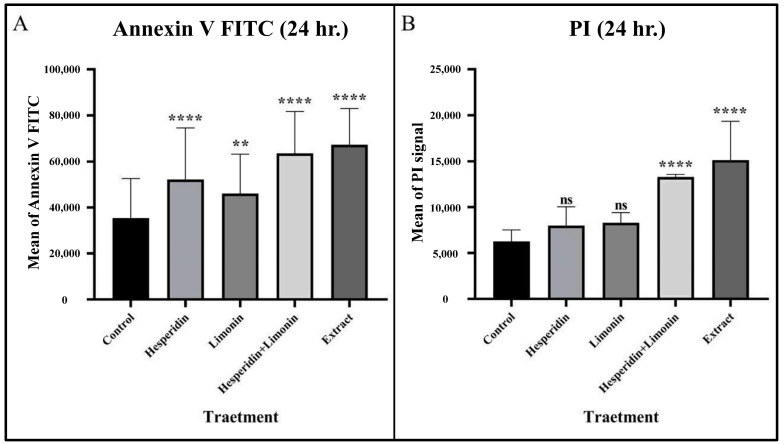
The mean and SD of Annexin V FITC (**A**) and PI (**B**) that stained in PLC/PRF/5 human hepatocellular carcinoma cells from flow cytometry analysis at 24 h in each treatment. The statistical differences were analyzed by using one-way ANOVA followed by the Bonferroni comparisons test. The symbol ns, ** and **** mean *p* > 0.05, <0.01 and <0.0001, respectively.

PLC/PRF/5 cells were treated with IC_50_ dose at 500 μg/mL, 165 μg/mL and 188 μg/mL for lime peel ethanolic extract, hesperidin, and limonin, respectively (0.5% DMSO was used to negative control). The percentage of early and late apoptosis and necrosis cells is shown in the right lower, right upper, and left upper quadrants of the figure, respectively. At 24 h, hesperidin, limonin, a combination of hesperidin and limonin, and lime peel ethanolic extract all significantly induced apoptosis. The percentage of early and late apoptosis cells increased from 3.4% and 0.4% in untreated cells to 9.0% and 2.2% in hesperidin-treated; 6.1% and 1.9% in limonin-treated; 26.3% and 7.8% in the combination-treated; and 21.9% and 9.8% in the extract-treated, respectively (shown in Figure 6). Compared to the control, a significant increase in the means of annexin V signal was seen for all treatments (shown in Figure 7A). However, only the combination and the extract treatments have significant increases in the means of PI signal (shown in Figure 7B).

**Figure 8:**
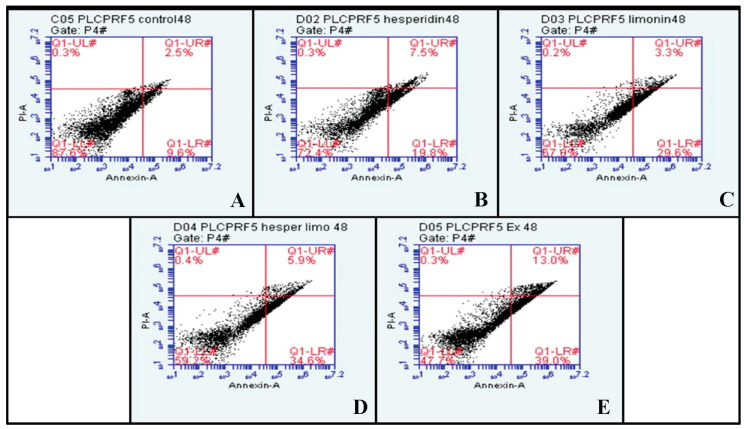
The dot plots of PLC/PRF/5 human hepatocellular carcinoma cells from flow cytometry analysis of Annexin V FITC/PI at 48 h in each treatment are (**A**) negative control (DMSO), (**B**) Hesperidin at IC_50_, (**C**) Limonin at IC_50_, (**D**) Combination of Hesperidin and Limonin at IC_50_, and (**E**) lime peel ethanolic extract at IC_50_.

**Figure 9:**
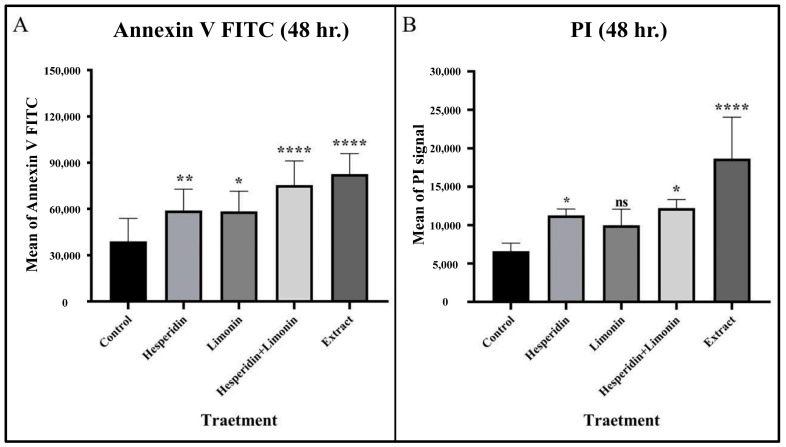
The mean and SD of Annexin V FITC (**A**) and PI (**B**) that stained in PLC/PRF/5 human hepatocellular carcinoma cells from flow cytometry analysis at 48 h in each treatment. The statistical differences were analyzed by using one-way ANOVA followed by the Bonferroni comparisons test. The symbol ns, *, **, and **** mean *p* > 0.05, < 0.05, <0.01, and <0.0001, respectively.

At 48 h, the percentage of early and late apoptosis cells increased from 9.6% and 2.5% in untreated cells to 19.8% and 7.5% in hesperidin-treated; 29.6% and 3.3% in limonin-treated; 34.6% and 5.9% in these combination-treated; and 39.0% and 13.0% in extract-treated, respectively (shown in Figure 8). Compared to the control, significant increases in the means of annexin V and PI signal were seen in almost all treatments except for PI in the limonin-treated condition (shown in Figure 9). 

**Figure 10:**
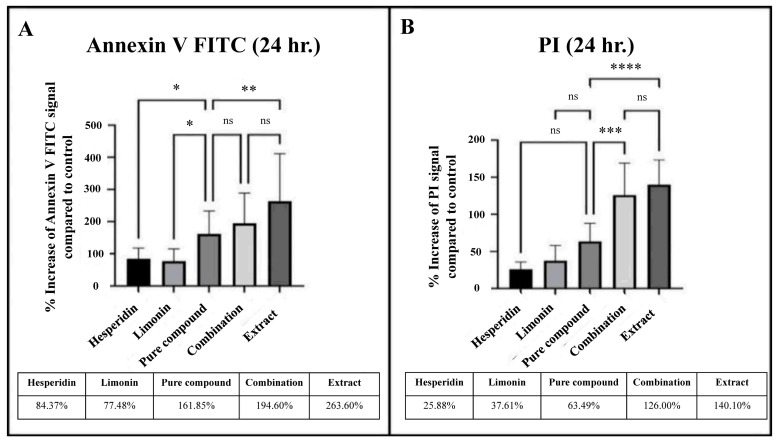
Percent increase of signal of Annexin V FITC (**A**) and PI (**B**) that stained in PLC/PRF/5 human hepatocellular carcinoma cells from flow cytometry analysis at 24 h in each treatment. The statistical differences were analyzed by using one-way ANOVA followed by the Bonferroni comparisons test. The symbol ns, *, **, *** and **** mean *p* > 0.05, < 0.05, <0.01, <0.001 and <0.0001, respectively.

From the comparison of the percent signal increase of Annexin V and PI staining, the percent cell death in lime peel extract treatment was significantly higher than those of the pure compounds (hesperidin or limonin treatment). However, when compared with the combination (hesperidin and limonin) treatment, it was found that although the treatment with lime peel extract had a higher tendency but not significantly different. Furthermore, we found the superior effect of a combination treatment was seen in both Annexin V and PI staining. The percentage of cell death in the combination treatment is more than the sum-up percentage of the single compounds (194.6% vs. 164.9% for Annexin; 126% vs. 63.49% for PI), although only PI staining showed statistical significance (shown in Figure 10). This result suggests a synergistic effect between limonin and hesperidin.

## 3. Discussion

Lime peel is the most abundant by-product from the citrus industry and a potential functional ingredient [19]. The health benefits of lime have been studied. However, most previous works either investigated phytochemicals in juice and essential oil sample or screened the groups of compounds only [20]. Here we reported the metabolomics profile of lime peel extract and its anti-cancer effects against liver cancer cells with p53 hotspot mutation PLC/PRF/5 cells. This study uncovers the novel findings that the ethanolic extract of lime peel is more potent than the pure limonin and hesperidin compounds in anti-cancer effects. Despite containing a many-fold lower amount of hesperidin and limonin, the lime peel extract at the IC_50_ level induced more apoptosis of PLC/PRF5 cells than that of pure limonin, pure hesperidin, or even a combination of pure limonin and pure hesperidin. Interestingly, we also found synergistic effects between limonin and hesperidin. Furthermore, the extract inhibited cancer cell invasion better than that of limonin but equal to that of hesperidin. Besides limonin and hesperidin, there were another 60 and 22 compounds detected from LCMS and GCMS analyses, respectively. Thus, the superior effect of the ethanolic extract against liver cancer cells compared to pure compound likely results from the combinatorial effects of limonin, hesperidin, and other phytochemical components in the extract. 

Metabolomics analyses of phytochemicals in lime peel’s ethanolic extract have not been reported before. Here we fill the gap of knowledge by using LC-qTOF/MS and GC-HRMS. These tools are powerful and robust because they allow simultaneous separation and identification of multiple compounds in the extract with accurate mass measurements and high resolution [21,22]. A previous study reported that the ethanolic extract of *Citrus aurantifolia*’s peel contains multiple groups of phytochemicals, including flavonoids, polyphenols, terpenoids, carotenoids, alkaloids, and glycosides [23]. However, that study did not perform metabolomics to identify specific names of compounds. Since there are no exactly similar published studies, we instead compare our results with the previous analytical studies of other kinds of citrus fruits. Previous studies of citrus fruit constituents (orange and bitter orange) reported a large, varied number of non-volatile compounds in citrus peel ranging from 14 to 142 components [24,25,26]. Our present study using LC-qTOF/MS in positive ion mode reveals 62 specific compounds in lime peel extract, which belong to multiple groups. The major components with the highest amount in the extract are coumarins (7-methoxy-coumarin and 5,7-dimethoxycoumarin), furanocoumarins (bergaptol, bergamotin), limonoids (limonin), and flavonoids (hesperidin and neohesperidin). Consistent with our study, a previous HPLC study of flavonoids in citrus constituents identified hesperidin as the highest flavonoid found in the peel of *Citrus aurantifolia* [11]. 

It is worth noting that some compounds reported in other citrus fruits [24,25,26] did not appear in our study. Besides, the different species of lime from other citrus fruits, the distinct extraction and analytical methods, source of raw materials, and preparation method for raw materials (e.g., drying process) may also contribute to the distinct [27,28]. For example, carbohydrates and various fatty acids reported in whole lime fruit from a previous study [26] do not appear in our LC-qTOF/MS metabolomics analysis of lime peel extract in this study. The difference could be due to either the different composition between whole fruit and peel or the sample preparation technique. Since this study did not do derivatization prior to scanning, some small molecules, such as short-chain fatty acids, may not be detected. Interestingly, some flavonoid glycosides such as Kaempferol, didymin, and their derivatives were found in other citrus fruits but not found in the ethanolic extract of lime peel in this study. The data suggests that lime may not be the source of these compounds, or the compounds may be degraded or rearranged during the manufacturing process [28,29]. 

Previous studies reported a large, varied number of volatile compounds ranging from 15–38 components in the essential oil of lime (*Citrus aurantifolia*), tangerine (*Citrus nobilis*), sweet orange (*Citrus sinensis*), lemon (*Citrus limon*), and Kaffir Lime (*Citrus hystrix*) [27,28,30]. Our present metabolomics study for ethanolic extract of lime peel using GC-HRMS discovered 22 volatile compounds, which is more than what had been reported in the essential oil of lime [28]. Similar to the previous studies [27,28], D-Limonene is the most abundant volatile compound found in the peel extract and essential oils of lime and other citrus fruits. While a previous study identified ꞵ-pinene as the second most abundant volatile in essential oil, we found only a small amount of α-pinene in the lime peel’s ethanolic extract. The difference is likely due to the different chemical properties between the two pinenes. While α-pinene is soluble in oil and ethanol, ꞵ-pinene is soluble in oil but not in ethanol or water [31]. These could explain why the ꞵ-pinene was found only in the essential oil but not in the ethanolic extract of our study. Interestingly, we discovered aromandendrene, isopimpinellin, and cis-α-bergamoten, which were not found in the GCMS analysis of lime peel oil in the previous report [28]. 

In general, the biological activity of medicinal plants/natural products is related to their chemical constituents. These chemical constituents could demonstrate their possible potential in preventing or treating cancer and other chronic diseases of medicinal plants/natural products [32]. Several compounds found in lime peel’s ethanolic extract of this study, including limonin, limonene, hesperidin, neohesperidin, hesperetin, bergaptol, and bergamottin were reported to have anti-cancer properties [33,34,35]. Among these compounds, hesperidin and limonin are most well-evidenced for anti-cancer effects [12,13,14,15,16,17]. Therefore, our previous study developed a green extraction method to obtain a hesperidin-limonin-rich ethanolic extract of lime peel. In this study, we compared the anti-cancer effect of the lime peel’s ethanolic extract with those of pure limonin and hesperidin. Future studies are warranted to study the combinatorial effects of other compounds with less known effects, such as bergaptol and bergamottin.

In this research, PLC/PRF/5 human hepatocellular carcinoma cell line was used to investigate the anticancer effects of the standard compounds and extracts. The cell line is a well-characterized hepatocellular carcinoma containing hotspot p53 mutation at R249, which is a unique hotspot codon of liver cancer [6,36]. Though previous studies have reported the effect of hesperidin or limonin on HepG2 cells, liver cancer cell lines with wild-type p53 gene [16,17], this is the first study to report the effect of hesperidin, limonin, combination, and the ethanolic extract of the lime peel on liver cancer cells with hotspot mutated p53. Interestingly, we found that at the IC_50_ level of the extract (according to MTT assay), the concentrations of hesperidin and limonin in lime peel extract are 580 and 13,628 times less than the IC_50_ of pure hesperidin and limonin, respectively. Consistently, apoptosis assay showed that the extract at the IC_50_ level induced a higher percentage of apoptosis than those of pure compounds or their combinations. The finding suggests that the extract is more potent than the pure compounds. Furthermore, we discovered the synergistic anti-cancer effect between limonin and hesperidin, which could be due to either the direct interaction or the synergistic mechanisms. In fact, previous studies reported that hesperidin could increase the cellular uptake efficiency of some nutrients/compounds [37,38]. Interestingly, despite the lower concentration of hesperidin and limonin, the extract still showed a significantly better effect than those of the single compounds and had a trend better effect than the combinatory hesperidin-limonin treatment, although it was not significantly different. This finding indicates that besides hesperidin and limonin, other compounds in the extract, such as limonene, neohesperidin, hesperetin, bergaptol, bergamottin, nomilin, synephrine, α-pinene, etc., may also play a role. In addition, these compounds in the extract may interact with each other, whether the combinatorial effect (additive or synergistic) or antagonistic effect. Future studies are warranted to elucidate the interaction of these compounds to understand and apply them to improve the treatment for better results. In this study, we validated the synergistic effect between hesperidin and limonin by comparing the effect of the combination with those of the single compound. Likewise, future studies shall quantify the amount of those other compounds in the extract and study the combinatorial effects of those compounds against cancer in comparison with those of the single compound. Alternatively, one could use computer modeling to predict the possible combination effect based on the structure and biological activity of those compounds [39]. 

It is worth noting that although the cells were treated at the IC_50_ levels derived from the MTT assay, the percentage of cell death was less than 50%. MTT assay depends on metabolic activities, and the loss of signal could occur either from cell cycle arrest or cell death [40]. Therefore, the IC_50_ doses from the MTT assay may not come solely from cell death inductive effect but rather from both cell death and cell cycle arrest. Future studies measuring changes in cell cycle states of PLC/PRF/5 cells after exposure to the lime peel extract, hesperidin, limonin, and the combinations are warranted to confirm such a hypothesis. 

The induction of apoptosis and cell cycle arrest and the suppression, invasion, and metastasis are all controlled by p53 [41,42]. Mutations of p53, such as that which appeared in PLC/PRF/5 human hepatocellular carcinoma cells, result in the loss of p53 functions, leading to abnormalities in cell death and cell cycle arrest and allowing invasion and metastasis of cancer cells [6,38]. Therefore, our result of cell death induction and invasion suppression in p53 mutated cells suggests that the phytochemicals in lime peel extracts may re-activate mutant p53 to restore wild-type p53 function. In fact, previous studies reported the p53 reactivation effect of hesperidin and limonin in p53-mutated ovarian cancer [37,43,44]. However, the underlying mechanism is unknown. Interestingly, previous works showed that restoration of wild-type p53 functions requires interaction between plakoglobin (PG, γ-catenin) and mutant p53 protein, which resulted in inhibition of invasion and metastasis of cancer cells [45,46]. Thus, future studies are warranted to test whether limonin, hesperidin, and the lime peel extract could reactivate p53 by promoting the interaction between p53 and plakoglobin (PG, γ-catenin). Besides the reactivation of p53, the induction of p63 and p73 is another possible pathway. The p63 and p73 are apoptotic-inducing proteins that are rarely lost or mutated in cancers [47,48]. Therefore, further studies should investigate the mechanistic pathways of apoptosis induction by hesperidin, limonin, and lime peel extract in p53-mutated hepatoma cells. 

It is worth noting that in this metabolomics study, we also found furan in the lime peel ethanolic extract. Furan is classified as group 2B (possibly carcinogenic to humans) according to International Agency for Research on Cancer (IARC) [49]. Previous work defined a benchmark dose level 10 (BMDL10) of 1.23 mg/kg/day for liver cancer and the margin of exposure (MOE) at 750 and 4300 for infants and adults, respectively [50]. Since our current study is a qualitative study, a future study is warranted to quantify the exact amount of furan in the lime peel extract. Then, a risk assessment to estimate the exposure compared with the reference dose should be performed to identify if there are any safety concerns. The appearance of furan in the extract could be either naturally occurring or from storage or the extraction process. A previous study in China found furan in the peel of mandarin oranges that had been picked in October month (autumn season) but not in other seasons [51]. Other studies showed that furan could be converted from a number of precursor compounds, including ascorbic acid, amino acid (e.g., serine, aspartic acid, threonine, and alanine), carbohydrate (e.g., ribose, xylose, arabinose, galacturonic acid), polyunsaturated fatty acids, and carotenoids [52,53,54]. Interestingly, in our current study, ascorbic acid is not detectable in the qualitative analysis by LC-qTOF/MS. Thus, it is possible that the furan and furfural we found may be formed by the degradation of ascorbic acid in lime peel. Previous studies reported that temperature and time in processing and storage could promote the conversion of ascorbic acid to furan and derivatives [55,56]. Therefore, to identify the source of furan in the lime peel extract, a further study should compare the amount of furan before and after each step of processing and extraction. 

The strength of this work includes the use of LC-qTOF/MS and GC-HRMS to investigate the metabolomics of the phytochemical profile in this study. It allows for accurate and specific separation and identification of the compounds. For the cell culture experiment, we chose PLC/PRF/5, a well-characterized hepatocellular carcinoma, to study. Also, we studied the extract, the pure limonin, pure hesperidin and also its combination. Nevertheless, the limitations of this work include the lime peel powder used for the preparation of the extract for this study was randomly collected from the same batch of industrial food waste. Future work on various batches of tests would be worthwhile. Based on numerous previous studies [24,25,26,27,28], the known compounds found in citrus fruits are mostly detectable in positive ion mode. Therefore, this study only scanned for compounds in the extract using positive ionization mode. Nevertheless, there could be some unknown compounds that might be detectable in negative ionization mode. Future studies should scan in both modes for a complete investigation. Furthermore, in this study, the LC-MS/MS screening was performed with a spike of 15% methanol during 21.00–21.10 min, which could be the reason why we observed multiple compounds eluting at 22 min. Future studies should try using a linear gradient from 1 to 100% MeOH at 30 min without spiking for better separation. For cell culture experiments, we chose only one dose, which is the maximum non-toxic dose at 24 h. for invasion assay. Future studies with various doses and times would be interesting. In this work, we investigated the anti-cancer effect of the lime peel’s ethanolic extract in the liver cancer cells with hotspot p53 mutation, in comparison with those of pure hesperidin and limonin compounds. The promising effect of the lime peel extract warrants further investigation for its selectivity (in comparison with normal liver cells) and efficacy (in comparison with chemotherapeutic drugs for liver cancer, such as Gemcitabine, Cisplatin, and Doxorubicin. 

## 4. Materials and Methods

### 4.1. Chemicals and Reagents

Lime peel powder was obtained from Chiangmai Bioveggie Co., Ltd. (Chiang Mai, Thailand). The powder was produced by vacuum drying fresh lime peel, which is food waste from the GMP-certified lime juice factory. Limonin (≥95.0% purity) and hesperidin (≥90.0% purity) were purchased from the Tokyo chemical industry (Tokyo, Japan). Eagle minimum essential medium (EMEM), fetal bovine serum (FBS), Phosphate-buffered saline (PBS) pH 7.4, 0.25% trypsin + 0.03% EDTA solution, penicillin/streptomycin, and Geltrex LDEV-free reduced growth factor were purchased from Gibco by Invitrogen Life Science Technologies, Thermo Fisher Scientific Inc. (Waltham, MA, USA). Dimethyl sulfoxide (DMSO), 3-(4,5 dimethylthiazol-2-yl)-2,5-diphenyltetrazolium bromide (MTT dye), were purchased from Sigma-Aldrich (St. Louis, MO, USA). Annexin V-FITC apoptosis detection kit with propidium iodide (PI) from Immune tools (Friesoythe, Germany). 

### 4.2. Preparation of Extract

Lime peel powder was extracted following the optimum condition method from a previous study [18]. Lime peel powder was extracted with 80% ethanol in water at pH 7 in a 50 °C shaking water bath (Memmert GmBH Co., Büchenbach, KG, Germany) at a shaking speed level of 3.5 for 100 min. The solid-to-solvent ratio was 0.01 g/mL. The extract was concentrated with a vacuum rotary evaporator and dried with a freeze-dryer. It was kept in a freezer at −20 degrees Celsius until for use in analysis and study in the cell culture model. 

The lyophilized extract was thawed and dissolved in DMSO prior to further dissolving in culture media. The final concentration of DMSO was no more than 0.5%, which was non-toxic to PLC-PRF5 according to our test. 

### 4.3. Metabolomics Analysis 

The lyophilized powder of lime peel’s ethanolic extract was dissolved in 50% DMSO: water (*v*/*v*) and filtered through a 0.2 µm nylon filter. Then, it was further diluted in water to yield a final concentration of 0.1% DMSO prior to LC-MS/MS or GC-HRMS analyses.

#### 4.3.1. Using LC-MS/MS 

The qualitative screening method of phytochemical in lime peel ethanolic extract was performed with high-performance liquid chromatography (HPLC)-with tandem quadrupole time-of-flight (Q-TOF) mass spectrometer by using TripleTOF^®®^ 6600+ quadrupole time-of-flight mass analyzer (AB SciEx, Framingham, MA, USA). The following method was modified from a previous study [24].

HPLC was performed with Gradient program by using Acclaim^TM^ RSLC 120 C18 column (100 mm × 2.1 mm, 2.2 µm particle size, pore size 120 Å, Thermo Fisher Scientific, Waltham, MA, USA) at a flow rate of 0.4 mL/min for 30 min. The column was maintained at 40 °C. The gradient run using 2 mobile phases consisted of 0.1% formic acid in water (A) and 0.1% formic acid in acetonitrile (B). It was performed as follows: 0.00–1.00 min, 1–1% B; 1.00–21.00 min, 1–80% B; 21.00–21.10 min, 80–95% B; 21.10–25.00 min, 95–95% B; 25.00–25.10 min, 95–1% B, 25.10–30.00 min, 1–1% B. The total run time of this method was 30 min/injection for the injection volume of 2 µL.

Mass spectrometric analysis was performed in positive ion full scan mode with a mass range of 100–1000 m/z. The following experimental parameters were used: spray voltage of 55 kV, source temperature of 500 °C, nebulization gas pressure of 50 psi, drying gas pressure of 60 psi, decluttering potential of 80 V, and collision energies of 30 ± 15 eV. Their MS spectra were compared with the mass spectral database of the national institute of standards and technology (NIST) and the natural products high-resolution MS/MS Spectral Library on SCIEX OS (AB SciEx, Framingham, MA, USA) to identify the phytochemical components.

We used the LC-QTOF/MS machine at Mahidol University Frontier Research Facility. Therefore, we optimized the method according to the in-house method suggested by the expert scientist of the core facility, who have long-term experience with the machine.

#### 4.3.2. Using GC-HRMS Analysis

The method to identify the volatile phytochemical components in lime peel ethanolic extract was modified from a previous study [57,58]. It was performed by using Orbitrap™ Exploris™ gas chromatography coupled with a high-resolution, accurate mass-mass spectrometer (GC-HRAM-MS) (Thermo Fisher Scientific, Waltham, MA, USA).

The GC-HRAM-MS system was equipped with a TG-5SilMS capillary column (thickness of the film: 0.25 µm, diameter: 0.25 mm, length: 30 m). The carrier gas (helium gas, 99.999% purity) flow rate at 1 mL/min. The injector temperature was 250 °C (constant), and the injection volume of 1 µL (split ratio = 50). The column over temperature was ramping performed with a thermal gradient program that set at 40 °C (hold for 1 min), a ramp of 10 °C/min to 50 °C/min (hold for 1 min), a ramp of 10 °C/min to 60 °C/min (hold for 1 min), a ramp of 10 °C/min to 160 °C/min, a ramp of 5 °C/min to 210 °C/min (hold for 1 min), and the final temperature was a ramp of 10 °C/min to 280 °C/min (hold for 10 min). The total run time in this method was 43 min/injection. Transfer line temperatures were set at 250 °C. Filament on at 3 min after injection (solvent delay). Electron ionization energy was 70 eV in full scan mode with the scan mass range of 40 to 600 m/z at a resolution of 60,000. Their MS spectra were compared with the mass spectral database of the national institute of standards and technology (NIST) on Freestyle software (Thermo Fisher Scientific, Waltham, MA, USA) to identify the phytochemical components.

To optimize the measurement, we performed a set-up trial by starting with an exactly similar method as the published references. Then, the parameters were adjusted to allow the maximum and clearest separation of the peaks. The oven temperature was ramped to the maximum resistance of the column (following the manufacturer’s guide). And the run time was held for the longest period to ensure that no component remained in the column.

### 4.4. Quantitative Measurement of Hesperidin and Limonin

The concentration of hesperidin and limonin in the dissolving extract was quantitatively measured by using LC-MS/MS-based method as described previously [18]. The method has been validated with satisfactory intra- and inter-day precision, linearity, and recovery [18].

### 4.5. Cell Culture

PLC/PRF/5, a human hepatocellular carcinoma cell line (the Alexander cell) containing the hepatitis B virus (HBV) genome, was obtained from American Type Culture Collection (ATCC) (Manassas, VA, USA). Cells were cultured in EMEM supplemented with 10% heat-inactivated FBS and 100 U/mL penicillin, and 100 µg/mL streptomycin at 37 degrees Celsius and 5% CO_2_ in an incubator. The cultured cells were routinely monitored under an inverted microscope, sub-cultured and the culture medium was replaced twice per week.

### 4.6. Cell Viability Assay 

The cell viability assay was studied by an MTT assay that was adapted from a previous study [59]. PLC/PRF/5 cells were seeded on a 96-well plate following the density from preliminary (1.2 × 10^5^ cell/mL) at 100 μL/well. Cells were treated with lime peel ethanolic extract, hesperidin, or limonin at various concentrations and incubated at 48 and 72 h. After incubation, the medium was removed, and cells were washed with PBS pH 7.4. Then, MTT dye solution (5 mg/mL in PBS) was added at 50 μL/well and incubated for 3 h at 37 degrees Celsius. Finally, the MTT dye solution was removed, and the violet crystal was dissolved in DMSO at 150 μL/well. It was used to determine the absorbance by microplate reader spectrophotometer at 570 nm and calculated cell viability by % of absorbance of treated cells to absorbance of negative control cells (0.5% DMSO treated).

### 4.7. Cell Invasion Assay 

The invasion assay was studied using a Transwell invasion assay [60], which was performed with some adaption from the manufacturer’s protocol. Transwell inserts for 24-well plates were a polyethylene terephthalate filter containing 8.0 µm pore size and coated with Geltrex™ at 50 μL/insert. The lower chamber was filled with 10% FBS medium. PLC/PRF/5 cells were seeded on the upper insert following the density from preliminary (1 × 10^5^ cell/mL) at 200 µL/insert. Cells were treated with the non-toxic concentration of lime peel ethanolic extract, hesperidin, or limonin in a free-FBS medium. After incubation at 24 h, the medium was removed and washed cells with PBS pH 7.4 (2×). Fixed the cells with 4% paraformaldehyde for 10 min, followed by staining with crystal violet for 20 min. Subsequently, removed the non-invasion cell and gel in the upper inserts were with a cotton swab. Invading cells were investigated and counted in three randomly selected fields under EVOS™ XL inverted microscope. The percent of invasion rate was calculated by the number of treated cells to the number of negative control cells (0.5% DMSO treated).

### 4.8. Cell Apoptosis Assay 

The cell apoptosis assay was studied by flow cytometric analysis of Annexin V/PI as described [61]. PLC/PRF/5 cells were seeded on a 6-well plate following the density from the preliminary (6 × 10^4^ cell/mL) at 3 mL/well. Cells were treated with the IC_50_ concentration (obtained from the MTT assay) of lime peel ethanolic extract, hesperidin, limonin, or the combination of limonin and hesperidin (both of IC_50_ concentration) (0.5% DMSO for control) and incubated at 24-, 48-hr. After incubation, cells were harvested and wash cells with PBS pH 7.4. Then, binding buffer (1×) was added at 94 μL and followed with Annexin V-FITC at 3 μL. It was incubated at room temperature for 15 min (protect from light). After that, PI was added at 3 μL and adjust the final volume to 300 μL with binding buffer. Cell death (apoptosis and necrosis) was determined by flow cytometric analysis of Annexin-PI within 1 h after staining.

### 4.9. Statistical Analysis

Statistical analyses were performed using GraphPad prism V.9. One-way analysis of variance (ANOVA) with Bonferroni comparisons test post hoc test was performed to compare the mean from at least 3 independent experiments of each treatment. IC_50_ values were identified from a regression equation obtained by a fitted non-linear regression model. A *p*-value < 0.05 indicated a statistically significant difference.

## 5. Conclusions

This study demonstrated that lime peels obtained as industrial food waste from lime juice factories are a potential source of raw materials for the extraction of natural products with potential health benefits. LC-qTOF/MS analysis demonstrated that the lime peel ethanolic extract contains at least 62 components, including the compounds in the glycoside, saccharide, amino acid, organic acid, alkaloid, flavonoid, flavonoid glycoside, furanocoumarin, and terpenoid (limonoids) groups. While GC-HRMS analysis demonstrated that the volatile compound in lime peel ethanolic extract contains at least those 22 components, especially the compound terpene groups. Lime peel extract has the potential to inhibit cell proliferation and induce apoptosis of p53 mutated liver cancer cells, PLC/PRF/5. The non-toxic dose of hesperidin, limonin, and lime peel extract can inhibit the invasion abilities of PLC/PRF/5 cells. Furthermore, this study discovered that the lime peel extract has a better effect on apoptosis induction than those of the pure compounds of hesperidin and limonin. In addition, the synergistic effect of hesperidin and limonin is observed for apoptosis induction and provides a good explanation for the superior effect of the extract. Since the effect of the extract is still better than the combination between hesperidin and limonin, the anti-cancer effect of the extract likely comes from not only the synergistic effect between hesperidin and limonin but rather the combinatorial effects of several other compounds detected in the metabolome.

This novel insight could have potential application for further investigation in animal models of liver cancer. Also, future studies in other cancer models with p53 mutation are also worthwhile. 

## Figures and Tables

**Table 1:** Identification of chemical components of lime (*Citrus aurantifolia*) peel ethanolic extract using HPLC-qTOF/MS.

No.	RT (min)	Compounds	Mode	Molecular Mass	Precursor Mass	Molecular Formula	Library Score	Relative Area (%)
1.	0.93	D-Aspartic acid	M^+^	133.0382	134.0449	C_4_H_7_NO_4_	90.3	0.10
2.	0.97	N-Oxalylglycine	[M + CH_3_OH + H]^+^	115.0272	148.0602	C_4_H_5_NO_5_	100.0	0.06
3.	1.05	Benzothiazole, 2-methylmercapto-	M^+^	181.0507	182.0575	C_8_H_7_NS_2_	99.2	0.04
4.	1.05	Melibiose	[M + NH_4_]^+^	342.1164	360.1497	C_12_H_22_O_11_	86.3	0.10
5.	1.20	3-Pyridinecarboxaldehyde	M^+^	107.0378	108.0445	C_6_H_5_NO	85.7	0.10
6.	1.28	Synephrine	M^+^	167.0588	168.0655	C_9_H_13_NO_2_	83.7	0.14
7.	1.57	2,5-Dihydroxy benzaldehyde	M^+^	137.9961	139.0028	C_7_H_6_O_3_	82.3	0.08
8.	1.57	trans-Aconitic acid	M^+^	174.0170	175.0237	C_6_H_6_O_6_	72.1	0.85
9.	1.57	Citric acid	[M + NH_4_]^+^	192.0279	210.0611	HOC (CH_2_CO_2_H)_2_	76.3	0.64
10.	3.34	Adenosine	M^+^	267.0976	268.1044	C_10_H_13_N_5_O_4_	100.0	0.40
11.	4.14	2,3-Desisopropylidenetopiramate	M^+^	321.0845	322.0913	C_9_H_17_NO_8_S	89.3	0.30
12.	4.28	Phenprobamate	M^+^	165.0794	166.0861	C_10_H_13_NO_2_	99.7	0.22
13.	5.30	Poly-L-asparagine	M^+^	114.1026	115.0387	C_4_H_6_ H_2_O_2_	98.9	0.03
14.	6.49	Stachydrine	M^+^	143.1732	144.0799	C_7_H_13_NO_2_	83.6	0.02
15.	7.32	Isoorientin	M^+^	448.1002	449.1069	C_21_H_20_O_11_	99.1	0.04
16.	7.40	Lactobionic acid	M^+^	380.1087	381.1155	C_12_H_22_O_12_	97.9	0.07
17.	7.83	Engeletin	M^+^	435.1177	435.1244	C_21_H_22_O_10_	80.0	0.06
18.	7.91	trans-3-Coumaric acid	[M + H]^+^	146.0374	147.0441	C_9_H_7_O_3_	96.9	0.25
19.	7.91	cis-3-Coumaric acid	M^+^	164.0476	165.0543	C_9_H_7_O_3_	80.1	0.05
20.	7.91	Quercetin	M^+^	302.0433	303.0501	C_15_H_10_O_7_	97.9	0.03
21.	7.99	Isovitexin	[M + H]^+^	432.1077	433.1144	C_21_H_20_O_10_	98.0	0.80
22.	8.11	Rutin	M^+^	610.4776	611.4843	C_27_H_30_O_16_	99.6	0.02
23.	8.54	Quercetin 3′-methyl ether	M^+^	316.0587	317.0654	C_16_H_12_O_7_	98.9	0.09
24.	8.54	Isorhoifolin	M^+^	578.1632	579.1699	C_27_H_30_O_14_	100.0	0.19
25.	8.54	Narirutin	M^+^	580.1779	581.1846	C_27_H_32_O_14_	92.0	0.09
26.	8.54	Narcissoside	M^+^	624.1702	625.1769	C_28_H_32_O_16_	97.9	0.66
27.	8.58	Bisdemethoxy curcumin	M^+^	308.0900	309.0967	C_19_H_16_O_4_	71.3	0.14
28.	8.83	Diosmin	M^+^	608.1755	609.1823	C_28_H_32_O_15_	99.9	0.62
29.	8.92	Hesperetin	M^+^	302.0799	303.0867	C_16_H_14_O_6_	99.5	0.69
30.	8.92	Hesperidin *	M^+^	610.1898	611.1966	C_28_H_34_O_15_	88.8	4.98
31.	8.92	Neohesperidin	M^+^	610.2029	611.2097	C_28_H_34_O_15_	82.4	4.29
32.	8.92	Hyperin	[M + CH_3_OH + H]^+^	432.1057	465.1387	C_21_H_19_O_12_	91.7	0.30
33.	9.32	Isorhamnetin 3-O-glucoside	M^+^	478.1113	479.1181	C_22_H_22_O_12_	98.9	0.11
34.	9.32	Syringetin 3-glucoside	M^+^	508.1228	509.1296	C_23_H_24_O_13_	93.8	0.57
35.	9.76	Mono-2-ethylhexyl phthalate	M^+^	278.1169	279.1237	C_16_H_21_O_4_	82.8	0.04
36.	9.84	Scoparone	M^+^	206.0261	207.0328	C_11_H_10_O_4_	92.5	0.06
37.	10.02	Heraclenin	M^+^	286.0855	287.0922	C_16_H_14_O_5_	95.0	0.68
38.	10.48	2′,6′-Dihydroxy-4- methoxychalcone-4′- O-neohesperidoside	M^+^	594.1943	595.2010	C_28_H_34_O_14_	79.3	0.04
39.	10.60	Oxypeucedanin hydrate	M^+^	304.1034	305.1102	C_16_H_16_O_6_	98.1	4.24
40.	10.83	Byakangelicol	M^+^	316.0955	317.1022	C_17_H_16_O_6_	93.0	0.45
41.	11.32	7-Methoxycoumarin	M^+^	176.0489	177.0556	C_10_H_8_O_3_	85.1	1.22
42.	12.07	Angelicin	M^+^	186.0316	187.0383	C_11_H_6_O_3_	98.3	0.03
43.	13.06	5,7-Dimethoxycoumarin	M^+^	206.0661	207.0729	C_11_H_10_O_4_	79.2	14.62
44.	13.38	Isopimpinellin	[M + NH_4_]^+^	246.0529	264.0862	C_13_H_10_O_5_	100.0	0.07
45.	13.98	Limonin *	M^+^	470.2057	471.2125	C_26_H_30_O_8_	95.3	5.01
46.	14.83	Nomilin	[M + H]^+^	514.2206	515.2273	C_28_H_34_O_9_	91.8	0.24
47.	15.88	Obacunone	M^+^	454.1990	455.2058	C_26_H_30_O_7_	86.1	0.03
48.	16.62	Monolinolenin (9c,12c,15c)	M^+^	352.2618	353.2685	C_21_H_36_O_4_	75.7	0.04
49.	18.98	1-Palmitoyl-2-hydroxy-sn-glycero-3-phosphoethanolamine	M^+^	453.2863	454.2931	C_21_H_44_NO_7_P	88.0	0.66
50.	19.47	1-Palmitoyl-sn-glycero-3-phosphocholine	M^+^	495.3338	496.3405	C_26_H_52_NO_8_P	94.4	1.40
51.	19.74	Esculetin	M^+^	178.0269	179.0336	C_9_H_6_O_4_	70.1	0.04
52.	20.08	1-Oleoyl-sn-glycero-3-phosphocholine	M^+^	521.3492	522.3560	C_26_H_52_NO_7_P	97.2	1.20
53.	20.63	Cinnamic acid	M^+^	148.0167	149.0234	C_9_H_8_O_2_	79.6	1.45
54.	21.13	8-Allyloxypsoralen	M^+^	242.0583	243.0650	C_14_H_10_O_4_	90.2	0.06
55.	21.17	3-Propyl- 2-methylpyrazine	M^+^	136.1255	137.1322	C_8_H_12_N_2_	81.3	0.49
56.	21.17	Xanthotoxol	M^+^	202.0275	203.0342	C_11_H_6_O_4_	95.3	1.20
57.	22.51	Bergaptol	M^+^	202.0350	203.0417	C_11_H_6_O_4_	85.8	14.30
58.	22.55	Bergamotin	M^+^	338.1583	339.1651	C_21_H_22_O_4_	82.2	8.35
59.	22.66	5-Geranoxy- 7-methoxy-coumarin	M^+^	328.1823	329.1890	C_20_H_24_O_4_	91.7	24.36
60.	23.61	8-O-Acetylharpagide	M^+^	406.0821	407.0888	C_17_H_26_O_11_	95.0	0.17
61.	23.75	Oleamide	M^+^	281.2732	282.2799	C_18_H_35_NO	76.9	2.39
62.	25.67	Hexadecyltrimethylammonium cation	M^+^	284.2878	284.2945	C_16_H_33_N(CH_3_)_3_	98.5	0.03

* Further confirmation in comparison with standard compounds.

**Table 2:** Identified compounds of lime (*Citrus aurantifolia*) peel ethanolic extract using GC-HRMS.

No.	RT (min)	Compounds	Molecular Mass	Molecular Formula	SI	RSI	Relative Area (%)
1.	3.0932	1,1-Diethoxyethane	118.1742	C_6_H_14_O_2_	622	884	1.06
2.	4.8921	Furfural	96.0841	C_5_H_4_O_2_	752	888	1.36
3.	7.1966	α-pinene	136.2340	C_10_H_16_	776	866	1.43
4.	7.2959	Furan	68.0740	C_4_H_4_O	849	911	9.35
5.	7.7484	5-Methyl furfural	110.1106	C_6_H_6_O_2_	803	905	1.71
6.	7.900	1-Methylcyclopentanol	100.1589	C_6_H_12_O_2_	689	792	2.04
7.	9.1655	D-Limonene	136.2340	C_10_H_16_	867	871	20.92
8.	9.7108	3-Carene	136.2340	C_10_H_16_	792	842	1.67
9.	11.8210	Terpinen-4-ol	154.2493	C_10_H_18_O	757	832	1.05
10.	12.0373	α-Terpineol	154.2493	C_10_H_18_O	817	897	1.99
11.	12.3772	5-Hydroxymethy furfural	126.1100	C_6_H_6_O_3_	736	758	4.59
12.	15.3505	Caryophyllene	204.3511	C_15_H_24_	888	896	7.60
13.	15.4631	Cis-α-Bergamotene	204.3511	C_15_H_24_	876	899	6.34
14.	15.8405	Aristolochene	204.3511	C_15_H_24_	762	835	1.71
15.	16.3901	α-Farnesene	204.3511	C_15_H_24_	733	747	6.31
16.	16.4895	Aromandendrene	204.3511	C_15_H_24_	867	881	8.61
17.	17.0126	6,9-Guaiadene	204.3511	C_15_H_24_	815	857	1.47
18.	17.0899	Selina-3,7(11)-diene	204.3511	C_15_H_24_	823	879	1.38
19.	24.1909	Citraptene	206.1947	C_11_H_10_O_4_	878	896	7.11
20.	25.5329	Bergapten	216.1895	C_12_H_8_O_4_	840	937	3.12
21.	28.3627	Isopimpinellin	246.2155	C_13_H_10_O_5_	860	873	8.43
22.	31.6207	Palmitic acid β-monoglyceride	330.5026	C_19_H_38_O_4_	667	789	0.95

Similarity index (SI) and Reverse similarity index (RSI) are: >900 is an Excellent Match, 800–900 is a Good Match, 700–800 is a Fair Match, and <600 is a poor match.

## Data Availability

Publicly available datasets were analyzed in this study. This data can be found here: https://docs.google.com/spreadsheets/d/1xfP4ifs4Kr1RwgkFlY0mYMRTKaaj99EFcvVHfqnhHmo/edit?usp=sharing (accessed on 10 February 2023).

## Sample Availability

Samples of the compounds are available from the authors upon reasonable request.
